# Supplementary material for: A data-driven investigation of human action representations
Source: Sci Rep. 2023 Mar 30;13:5171. doi: 10.1038/s41598-023-32192-5 (PMC10063663; doi:10.1038/s41598-023-32192-5)
Supplement: Supplementary file 2 — Supplementary Information 2. [file 41598_2023_32192_MOESM2_ESM.pdf]

Supporting Information

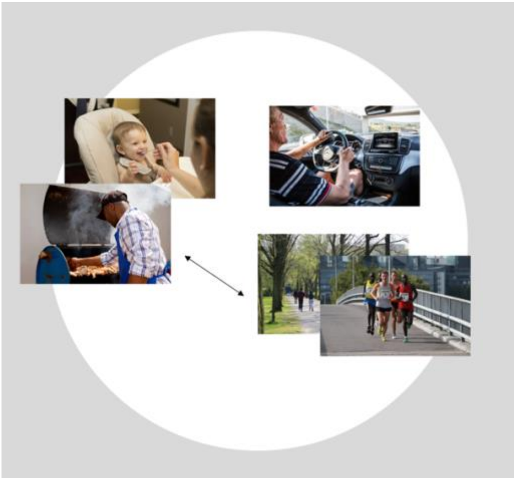

Supplementary Figure 1. Example of a multiple arrangement trial. The images are public domain examples of the types of actions included in the experiments.

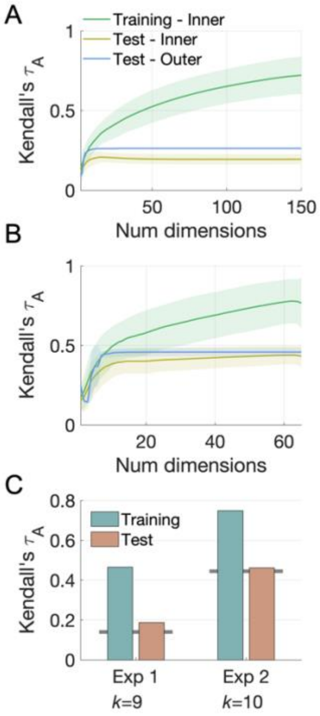

Supplementary Figure 2. NMF performance. **A.** Training set NMF reconstruction performance in Experiment 1 evaluated on the three sections of the training set (see Figure 6). Error bars are ±1SD. Performance on the outer set in the nested cross-validation procedure plateaus with nine dimensions. **B.** As in A, for Experiment 2. **C.** Final performance of the reconstructed matrix on the whole training set (~90% of the data) and the held-out set (~10% of the data), plotted against the true hold-out correlation (gray horizontal bars).

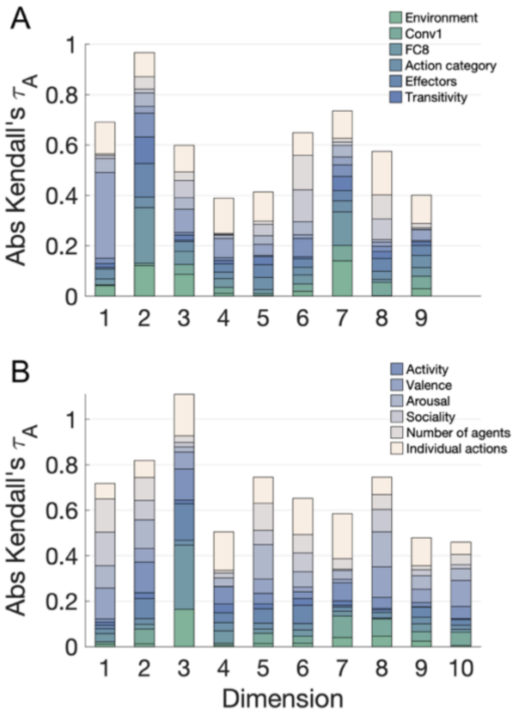

Supplementary Figure 3. Absolute correlations between visual, social, and action features and each NMF dimension for **A.** Experiment 1 and **B.** Experiment 2, shown in a stacked plot. Dimension are sorted in descending order of their summed weights.

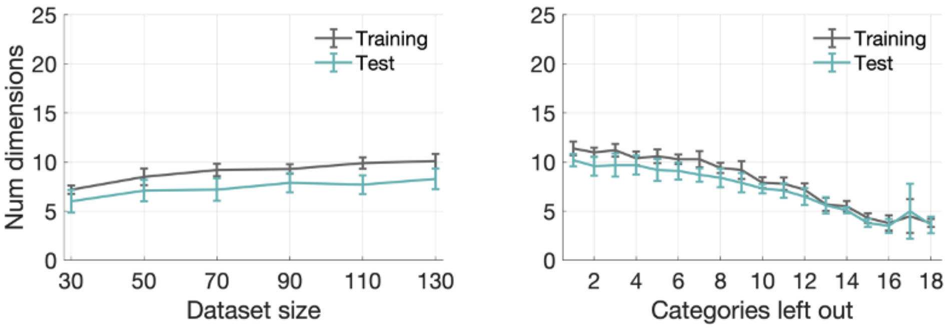

Supplementary Figure 4. Dimensionality as a function of dataset size. **Left**: number of dimensions obtained by running the NMF procedure using random subsets of the stimuli from Experiment 1 (10 iterations). **Right**: number of dimensions obtained by running NMF after leaving out a number of randomly selected action categories (10 iterations). The number of action categories has a larger effect on dimensionality than the dataset size.

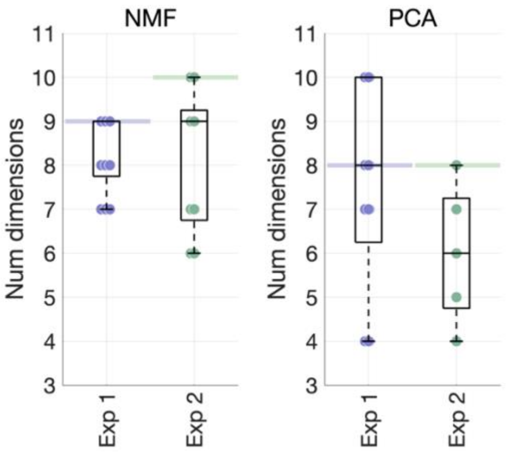

Supplementary Figure 5. NMF and PCA dimension robustness. The PCA procedure was repeated five times, after removing key stimulus categories from the behavioral RDM from Experiment 1**.** Each dot shows the number of dimensions resulting from each iteration, with horizontal lines showing the number of dimensions recovered from the full datasets.

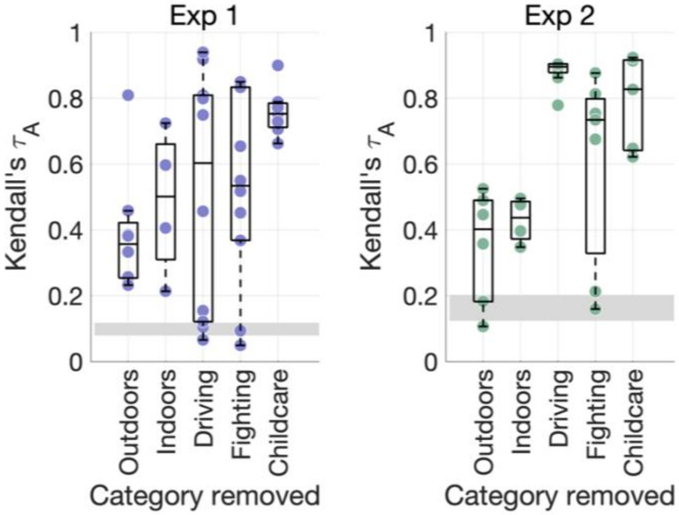

Supplementary Figure 6. PCA dimension robustness. The PCA procedure was repeated five times, after removing key stimulus categories from the behavioral RDM from Experiment 1**.** Each dot shows the maximal correlation between each dimension obtained in the control analysis and any of the original dimensions with the same stimuli removed (repeats allowed). The grey rectangles depict the chance level. Although on average correlations are higher than those obtained with NMF, their variance is overall almost twice as high, suggesting that stimulus set perturbations have a stronger impact on some of the PCA dimensions.

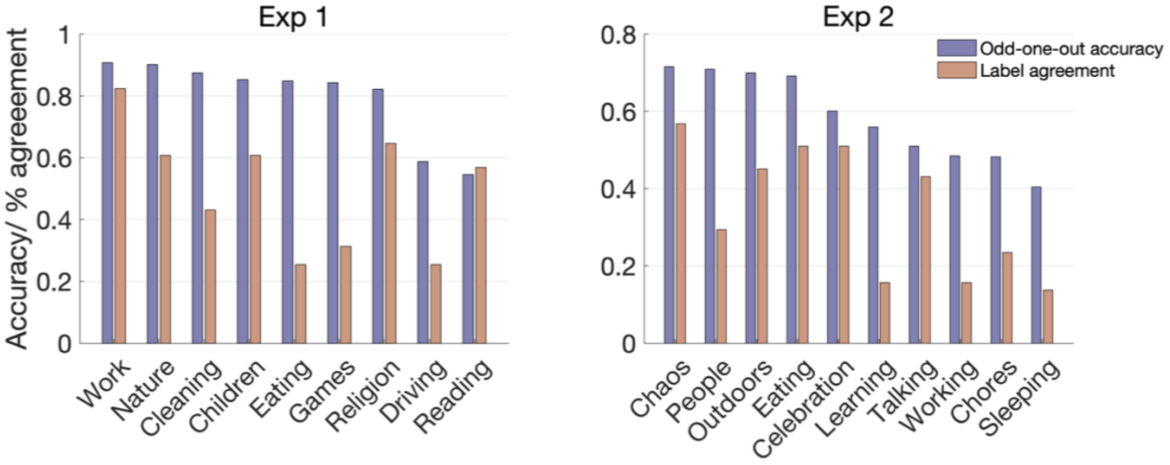

Supplementary Figure 7. Validation variability. Dimensions are named according to their most common labels, and ranked according to the accuracy obtained for each of them in the odd-one-out task. Participant agreeement on the most common label is also shown for each dimension.

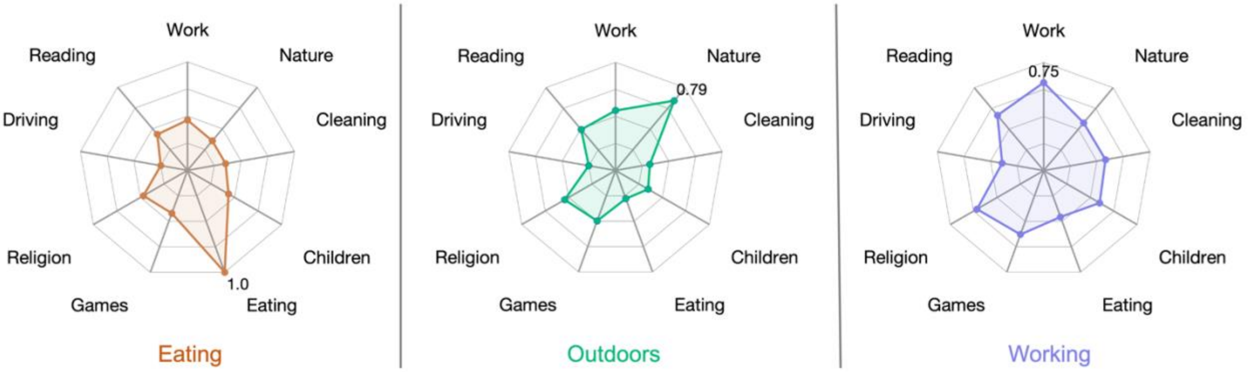

Supplementary Figure 8. Examples of how three dimensions from Experiment 2 map onto the dimensions from Experiment 1, as measured via semantic embeddings of the labels given by participants. The similarity values shown are relative (i.e. normalized to the 0-1 range).
